# Impact of removing prescription co-payments on the use of costly health services: a pragmatic randomised controlled trial

**DOI:** 10.1186/s12913-022-09011-0

**Published:** 2023-01-14

**Authors:** Pauline Norris, Kim Cousins, Simon Horsburgh, Shirley Keown, Marianna Churchward, Ariyapala Samaranayaka, Alesha Smith, Carlo Marra

**Affiliations:** 1 Va’a o Tautai- Centre for Pacific Health, University of Otago, PO Box 56, Dunedin, 9011 New Zealand; 2 Department of Preventive and Social Medicine, University of Otago, PO Box 56, Dunedin, New Zealand; 3 Turanga Health, 145 Derby St, Gisborne, 4010 New Zealand; 4 Health Services Research Centre, Victoria University of Wellington, PO Box 600, Wellington, New Zealand; 5 School of Pharmacy, University of Otago, PO Box 56, Dunedin, New Zealand

**Keywords:** Health services research, Randomised controlled trial, New Zealand, Prescription medicines, Co-payments

## Abstract

**Objectives:**

To determine whether exempting people (with high health needs and living in areas of high deprivation) from a $5 prescription charge reduces hospital use.

**Design:**

Two-group parallel prospective randomised controlled trial.

**Setting:**

People living in the community in various regions of New Zealand.

**Participants:**

One thousand sixty one people who lived in areas of high socioeconomic deprivation, and either took medicines for diabetes, took antipsychotic medicines, or had chronic obstructive pulmonary disease (COPD). Of the 1053 who completed the study, just under half (49%) were Māori.

**Interventions:**

Participants were individually randomized (1–1 ratio) to either be exempted from the standard $5 charge per prescription item for one year (2020-2021) (*n* = 591) or usual care (*n* = 469). Those in the intervention group did not pay the standard NZ$5 charge, and pharmacies billed the study for these. Participants continued to pay any other costs for prescription medicines. Those in the control group continued to pay all prescription charges for the year although they may have received one-off assistance from other agencies.

**Main outcome measures:**

The primary outcome was length of stay (hospital bed-days). Secondary outcomes presented in this paper included: all-cause hospitalisations, hospitalisations for diabetes/mental health problems/COPD, deaths, and emergency department visits.

**Results:**

The trial was under-powered because the recruitment target was not met. There was no statistically significant reduction in the primary outcome, hospital bed-days (IRR = 0.68, CI: 0.54 to 1.05). Participants in the intervention group were significantly less likely to be hospitalised during the study year than those in the control group (OR = 0.70, CI: 0.54 to 0.90). There were statistically significant reductions in the number of hospital admissions for mental health problems (IRR = 0.39, CI: 0.17 to 0.92), the number of admissions for COPD (IRR = 0.37, CI: 0.16 to 0.85), and length of stay for COPD (IRR 0.20, CI: 0.07 to 0.60). Apart from all-cause mortality and diabetes length of stay, all measures were better for the intervention group than the control group.

**Conclusions:**

Eliminating a small co-payment appears to have had a substantial effect on patients’ risk of being hospitalised. Given the small amount of revenue gathered from the charges, and the comparative large costs of hospitalisations, the results suggest that these charges are likely to increase the overall cost of healthcare, as well as exacerbate ethnic inequalities.

**Trial registration:**

Australian New Zealand Clinical Trials Registry (ANZCTR): ACTRN12618001486213 registered on 04/09/2018.

**Supplementary Information:**

The online version contains supplementary material available at 10.1186/s12913-022-09011-0.

## Background

In New Zealand and elsewhere, there are socio-economic and ethnic disparities in use of medicines [1–6], and these are likely to exacerbate inequities in health status. Compared to other barriers to accessing and using medicines appropriately (e.g. geographical distance from healthcare [7], patient knowledge and beliefs [8–10]), prescription co-payments are easily amenable to policy change. In New Zealand, co-payments are low ($5 per item with an annual ceiling of 20 items per family, after which co-payments are waived) but there are no exemptions for people with low income or severe chronic disease. There are also user charges for GP visits, which can be high[11]. Māori and Pacific people and those living in more deprived areas are at higher risk of being unable to afford their prescription medicines [12, 13]. Prescription co-payments were introduced in the mid-1980s [14], and we have not found a clear statement of the policy objective of co-payments. However, controlling government expenditure (through cost-sharing, and also through reducing ‘unnecessary’ use or wastage) appears to be a key goal [15].

Most studies of the impact of patient payments for prescription medicines are observational. These have found increasing co-payments can lead to lower use of medicines [16–18], poorer health status [19, 20] and increased use of other healthcare [18, 21, 22]. A Cochrane review [23] found only one randomised controlled trial, the Rand Health Insurance Experiment in the US in the 1970s, where people were randomly allocated to insurance programmes with differing levels of cost-sharing [24]. In the Rand study the effects of varying co-payments for medicines could not be isolated from the effects of co-payments for other care.

Existing studies rarely include the option of zero co-payments. Wales, Scotland and Northern Ireland have introduced free prescriptions, but quantifying the impact of this is complex, in part because most prescriptions (particularly those for people with high health needs or low income) were exempt from co-payments even before the change [25]. Studies which do include free prescriptions sometimes provide these as part of a package of interventions, making it difficult to tease out their impact (e.g. the CLEAN trial only provided medicines on an essential drugs list, and also mailed out medicines, reducing other barriers to access [26]). The MI FREEE cluster randomised trial was carried out with patients of one insurer, and focussed on one condition, only providing free medicines specific to the condition [27]. The aim of our study was to determine whether, for people at high risk of being unable to afford medicines, being exempted from the standard $5 prescription charge *for all medicines* reduced length of stay (hospital bed-days). In addition, we explored the impact on secondary outcomes of all-cause hospitalisations, hospitalisations for diabetes/mental health problems/COPD, deaths, and emergency department visits.

## Methods

The protocol for the study (known as the FreeMeds study) and the recruitment process are published elsewhere [28, 29].

This was a two-group parallel prospective randomised controlled trial. People living in areas of high socio-economic deprivation (NZDep 7–10) were eligible if they self-reported that they took medicines for diabetes, or anti-psychotic medication, or had COPD. NZDep is an area-level measure of deprivation, derived from census data such as income levels, damp and mouldy housing, internet access, and rental housing. NZDep 7–10 refers to the most deprived 40% of areas in NZ [30]. These criteria were selected as proxies for low income, high medication needs, and possibly requiring hospitalisation if medicines are not taken [31–35] rather than for special interest in these conditions. COPD and NZDep7 were added after 256 participants had been recruited, to increase the number of eligible people.

The study was carried out in several geographic areas of New Zealand, including small cities, towns and rural areas in both the North and South Island. No major metropolitan areas were included because a major supermarket chain offers free prescriptions at its in-store pharmacies in major cities.

Potential participants were informed about the study by pharmacy staff, Facebook, media stories, mail-drops, through general practices and community organisations. Participants were enrolled in person or on the phone, to provide a range of options and ensure that people who were disengaged from health services could also enroll.

At recruitment, participants provided demographic data and were randomized into intervention or control. Participants were randomly allocated 1:1 to either intervention or control group. There was no blocking on geographical location, and the allocation sequence was concealed from the recruiter. Those participants who enrolled in person were asked to tap an icon on a tablet computer which randomly turned red or green indicating that they were in the intervention or control group (“Randomizer – random generator” on android devices or “Coin Flipper” on PCs). Staff did this for those who enrolled on the phone and informed participants of their allocation. Blinding participants was not possible or desirable (they knew whether they paid for their prescriptions or not, and the security of knowing that they could access medicines without charge was an important aspect of the intervention). Similarly, community pharmacies could not be blinded. However, the researchers involved in the analysis of outcome data were blinded to participant allocation status.

The intervention was designed to mimic what might happen if the government decided to exempt this group from $5 prescription co-payments for all medicines they received (not just those for the conditions listed in the eligibility criteria), while the control group experienced usual practice for the study year (and were sent a $100 grocery voucher after the study period to thank them for their participation). There was no centralised system for indicating to pharmacies that participants were in the intervention group. Community pharmacies were given a list of all participants in the intervention group in their region, so that they could exempt participants from all $5 prescription co-payments for any medicines and instead invoice the study for these. Participants in the intervention group were also given a study ID card with their name, photo and NHI number, so they could visit any pharmacy and be exempted from the $5 charge. Participants continued to pay any additional charges, such as for blister packing. Participants in the control group continued with usual care, i.e. being charged $5 per prescription item until they reached the 20 item annual payment ceiling. Some may have accessed one-off support from charities etc. There were no pharmacies waiving the $5 co-payment in the study areas at the time of the study, but it is possible that some participants occasionally took advantage of these if they or others were travelling to other areas.

Recruitment had to occur in a narrow timeframe (October – February) because of the design of the copayment system in New Zealand. On 1 February each year co-payments commence, and after an individual or family is dispensed 20 items, copayments are waived. The intervention needed to start for all participants on or near 1 Feb to ensure that they got free prescriptions for a full year. If, for example, if it started in September many people in both intervention and control groups would have already reached the 20 item limit and so the intervention would have had no effect for several months. Recruitment therefore had to cease in early February. It was very difficult to recruit before the end of October because prescription charges were not a salient issue for many people at that time of the year and the intervention seemed too far in the future.

The primary outcome was length of stay (measured by hospital bed-days) within the year of the study (1 Feb 2020 to 31 Jan 2021). Secondary outcomes included: deaths (regardless of cause), ED visits (whether people visited ED during the year, and number of visits) and hospital admissions (whether people were hospitalised, number of hospitalisations and length of stay for diabetes, mental health and COPD).

NHI numbers for participants were determined on enrolment or soon after, and data on all of the outcomes presented here were accessed, with participants’ consent, from routinely collected data held by the Ministry of Health. Data on medicines dispensed are from the Pharms dataset. Data on hospitalisation outcomes, including diagnosis information and length of stay, were obtained from the National Minimum Dataset (NMDS) [36], which records discharges from public and private hospitals in New Zealand. Length of stay in this dataset is recorded as the number of midnights a person was in hospital for, so a person admitted and discharged on the same day would have a length of stay of zero. Deaths were identified using the National Health Index (NHI) collection [36], which contains demographic information about healthcare users including their dates of birth and death. Emergency department visits were identified using the National Non-Admitted Patient Collection (NNPAC) [36], which records outpatient events (such as visits to a specialist) and emergency department visits, and includes the date of the event.

The secondary cause-specific hospitalisation outcomes were identified using the International Statistical Classification of Diseases and Related Health Problems, Tenth Revision, Australian Modification (ICD-10-AM) diagnosis codes recorded in the NMDS and NNPAC. The specific approach used for each of the hospitalisation outcomes, as well as the ICD-10-AM codes used, are described in Supplementary Material. We included all hospitalisation for mental health problems since anti-psychotic medicines are commonly used for a wide range of mental health conditions [37, 38].

The only change made to trial outcomes after the study commenced was the addition of hospitalisations for COPD (because we expanded eligibility to those with COPD).

We planned to recruit 2000 participants in order to achieve a power of 80% to detect a 10% reduction in the primary outcome in the intervention group. Because enrolment was slower than anticipated we enrolled 1061 people.

No interim analyses were performed and no guidelines for stopping the trial were developed, because it seemed unlikely that the intervention, which aimed to ensure people could access medicines prescribed for them, would cause harm (although we acknowledge that harm can be inadvertently caused by access to participants’ data).

### Statistical methods

To assess the success of randomization of participants between intervention and control groups, and to identify possible residual confounding factors, participants’ socio-demographic characteristics at baseline were compared between two groups using chi-square test (or Fisher’s exact test as appropriate) to assess their comparability. Participants could indicate multiple ethnicities at enrollment, so for this comparison they were categorized into prioritized ethnic groups according to standard ethnicity protocols of the New Zealand Ministry of Health [39].

A small number of participants died during the year of follow-up, as a result their outcome measures were recorded in MOH databases over a shorter period than others. Occurrence of this happening was compared between two groups using Fisher’s exact test after allocating them to number of quarters they were followed.

As a preliminary assessment of the effectiveness of the intervention, we compared each outcome measure between two groups using chi-square test (or Fisher’s exact test as appropriate) after categorizing continuous outcomes.

The two main outcome measures were the length of stay (total number of midnights participants stayed in the hospital; LOS), and the number of hospital admissions during the study year. Both these were highly skewed; a large proportion of participants had zero or near zero outcomes while few participants had large outcomes. Therefore, we considered different types of statistical models (Poisson, negative binomial, zero inflated Poisson, zero inflated negative binomial). Based on the AIC (Akaike information criterion) and BIC (Bayesian information criterion), the negative binomial model was the best for these data. To account for the shorter follow-up period for those died within the year, we specified the follow-up period for each participant as the ‘exposure’ when using the negative binomial model.

To compare binary outcomes measures (i.e., admitted or not to hospital during the year, disease-specific as well as overall) between two groups we used logistic regression. Because we observed that rurality was likely to be unbalanced between the two groups (Table 1), and it is also likely be associated with the outcomes, we used rurality as an adjusting variable in all models.

**Table 1:** Baseline characteristics of participants in each study group

	**Intervention n (%)**	**Control n (%)**	**Total n (%)**	***P* value (chi-square or Fisher’s exact)**
Age, Mean (SD)	60.3 (14.0)	61.5 (13.8)	60.8 (13.9)	0.10
Males N (%)	262 (44.7)	194 (41.5)	456 (43.3)	0.30
**Ethnicity**^**a**^				
European	288 (49.1)	247 (52.9)	535 (50.8)	
Māori	302 (51.5)	211 (45.2)	513 (48.7)	
Pacific Peoples	14 (2.4)	10 (2.1)	24 (2.3)	
Asian	6 (1.0)	5 (1.1)	11 (1.0)	
Other	44 (7.5)	50 (10.7)	94 (8.9)	
Missing	0 (0)	3 (0.6)	3 (0.3)	
**NZDep**				0.49
1–6	16 (2.7)	18 (3.9)	34 (3.2)	
7–8	134 (22.9)	113 (24.2)	247 (23.5)	
9–10	436 (74.4)	336 (71.9)	772 (73.3)	
**Rurality**^**b**^**:**				0.07
Urban 1 (most urban)	0	0	0	
Urban 2	280 (47.8)	216 (46.3)	496 (47.1)	
Rural 1	227 (38.7)	170 (36.4)	397 (37.7)	
Rural 2	61 (10.4)	51 (10.9)	112 (10.6)	
Rural 3 (most rural)^b^	18 (3.1)	30 (6.4)	48 (4.6)	
**Medicines used 1 Feb 2019 – 31 Oct 2019**^**c**^				
Number of new dispensings per person	29.83	29.82		
Number of different medicines per person	12	12		

^a^Multiple ethnicities could be given so numbers do not add up to 100%. Therefore, a *P* value is not presented. We used prioritized ethnicity to examine the difference between groups, and *p* = 0.18

^b^Geographic classification for health (GCH) [40] 19% of the New Zealand population live in rural areas (1, 2 and 3)

^c^ “Before the study” is defined as 1 Feb 2019 to 31 October 2019. People start paying for 20 items at 1 Feb each year so this is an appropriate start date. After enrolling in the study the control group had an incentive to get as many medicines as possible before copayments started on 1 Feb 2020. Those in the intervention group had no such incentive, so to avoid any effect of the study on behaviour, we looked at the period before recruitment started

LOS was truncated to the end of the study (i.e. if someone was admitted on 25 Jan 2021 and was still in hospital at the end of the study on 31 Jan 2021, this was recorded as 6 days). A *p*-value less than 0.05 was considered statistically significant.

## Results

**Fig. 1:**
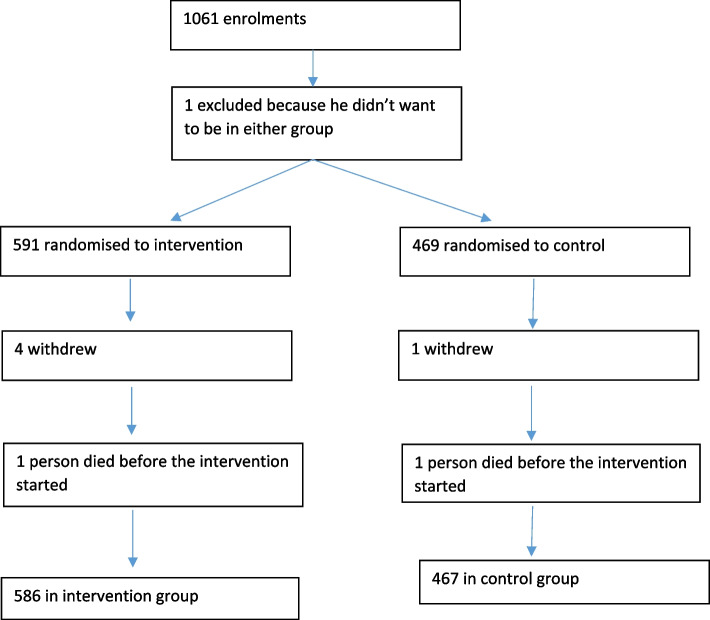
Participant flow

Of the 1061 enrollments in the study, one was excluded because he did not want to be randomized to a group, and a further five chose to withdraw. Two more were excluded because they died prior to the start of the intervention (see Fig. 1).

Recruitment was carried out 30/10/2019 – 7/2/2020. Participants in the intervention group were exempted from prescription co-payments 1/2/2020–31/1/2021 corresponding with the period in which items are counted to reach the 20 item payment ceiling.

Baseline characteristics of the participants are presented in Table 1. Data on medicines dispensed in the time period prior to the study are from the Pharms dataset. All other data in the table are based on self-report (rather than administrative data which is more likely to be out of date or inaccurate). Nearly half of our participants were Māori, and nearly three-quarters were in the two most deprived deciles. There were no substantial differences between the intervention and control groups. There were nearly significant differences in rurality, which is why we adjusted for this in subsequent analyses. ‘Number of new dispensings per person’ shows the average number of times a medicine was dispensed to people in the year before the study. Repeat prescriptions are excluded. Medicines use was almost identical between the groups.

Table 2 provides a summary of health-related outcomes for each group. During the year of follow-up, there were more deaths in the intervention group than in the control group (although this was not significantly different). All other outcomes were better for the intervention group than for the control group, but only whether or not admitted to hospital, mental health LOS, COPD LOS were statistically significantly different.

**Table 2:** Summary of health-related outcomes

Characteristic	Intervention		Control		Total		*P* value (chi-square or Fisher’s exact)
	**N**	**%**	**N**	**%**	**N**	**%**	
All	586	100	467	100	1053	100	
Died during the study	19	3.2	13	2.8	32	3.0	0.67
Visited ED	204	34.8	183	39.2	387	36.8	0.14
**Number of ED visits during the year**							
0	382	65.2	284	60.8	666	63.2	
1	96	16.4	80	17.1	176	16.7	
2–3	64	10.9	60	12.8	124	11.8	
4–7	33	5.6	35	7.5	68	6.5	
8 +	11	1.9	8	1.7	19	1.8	0.54
Mean (SD)	0.98 (2.77)		1.05 (2.16)		1.01 (2.52)		
**Admitted to hospital**	194	33.1	193	41.3	387	36.8	0.01
**Number of hospital admissions**							
0	392	66.9	274	58.7	666	63.2	
1	99	16.9	91	19.5	190	18.0	
2–3	62	10.6	66	14.1	128	12.2	
4–7	26	4.4	31	6.6	57	5.4	
8 +	7	1.2	5	1.1	12	1.1	0.07
Mean (SD)	0.77 (1.88)		0.97 (1.76)		0.86 (1.83)		
**Total LOS (days)**							
0 days	446	76.1	330	70.7	776	73.7	
1–2 days	55	9.4	41	8.8	96	9.1	
3–7 days	36	6.1	46	9.9	82	7.8	
8–14 days	23	3.9	18	3.9	41	3.9	
15–30 days	19	3.2	19	4.1	38	3.6	
31 + days	7	1.2	13	2.8	20	1.9	0.09
Mean (SD)	2.08 (6.86)		3.26 (9.78)		2.60 (8.30)		
**Admitted for Mental health conditions**	9	1.5	16	3.4	25	2.4	0.13
**Number of Mental Health admissions**							
0	577	98.5	451	96.6	1028	97.6	
1	8	1.4	13	2.8	21	2.0	
2 +	1	0.2	3	0.6	4	0.4	
Mean (SD)	0.02 (0.14)		0.04 (0.25)		0.02 (0.20)		
**Mental Health LOS (days)**							
0 days	579	98.8	452	96.8	1031	97.9	
1–2 days	4	0.7	4	0.9	8	0.8	
3 + days	3	0.5	11	2.4	14	1.3	0.03
Mean (SD)	0.11 (1.58)		0.21 (1.48)		0.15 (1.54)		
**Admitted for COPD**	17	2.9	24	5.1	41	3.9	0.06
**Number of COPD admissions**							
0	569	97.1	443	94.9	1012	96.1	
1	14	2.4	19	4.1	33	3.1	
2 +	3	0.5	5	1.1	8	0.8	0.17
Mean (SD)	0.03 (0.21)		0.09 (0.69)		0.06 (0.48)		
**COPD LOS (days)**							
0 days	573	97.8	445	95.3	1018	96.7	
1–2 days	3	0.5	9	1.9	12	1.1	
3 + days	10	1.7	13	2.8	23	2.2	0.049
Mean (SD)	0.13 (1.04)		0.53 (4.92)		0.30 (3.37)		
**Admitted for Diabetes**	8	1.4	12	2.6	20	1.9	0.16
**Number of Diabetes admissions**							
0	578	98.6	455	97.4	1033	98.1	
1	6	1.0	9	1.9	15	1.4	
2 +	2	0.3	3	0.6	5	0.5	0.36
Mean (SD)	0.03 (0.33)		0.04 (0.26)		0.03 (0.30)		
**Diabetes LOS (days)**							
0 days	579	98.8	456	97.6	1035	98.3	
1–2 days	2	0.3	4	0.9	6	0.6	
3 + days	5	0.9	7	1.5	12	1.1	0.26
Mean (SD)	0.15 (2.05)		0.18 (1.78)		0.16 (1.94)		

Table 3 presents incidence rate ratios of different outcomes estimated from negative binomial regression comparing intervention group to control group. Table 4 summarises the odds ratios for being admitted to hospital at least once during the study estimated using logistic regression.

**Table 3:** Summary of rurality adjusted estimates of the effect of the intervention on hospitalisations and length of stay from NBREG

	**Observed Incidence rate**		**Model predicted incidence rate**		**Adjusted Incidence rate ratio**		
Outcome measure	**Intervention**	**Control**	**Intervention**	**Control**	**IRR**^a^	**CI for IRR**	***P*-value**
Hospital admissions	0.773	0.966	0.817	1.01	0.81	0.626, 1.047	0.11
Total LOS	2.078	3.257	2.516	3.697	0.68	0.541, 1.027	0.07
Mental health admissions	0.017	0.043	0.017	0.044	0.39	0.169, 0.915	0.03
Mental health LOS	0.109	0.206	0.099	0.232	0.43	0.126, 1.452	0.17
COPD admissions	0.034	0.094	0.036	0.098	0.37	0.162, 0.853	0.02
COPD LOS	0.126	0.529	0.13	0.656	0.20	0.065, 0.60	*P* < 0.01
Diabetes admissions	0.027	0.036	0.029	0.033	0.88	0.29, 2.64	0.82
Diabetes LOS	0.147	0.18	0.177	0.146	1.21	0.268, 5.49	0.80
ED admissions	0.985	1.049	1.016	1.082	0.94	0.716, 1.232	0.65

^a^*IRR* incidence rate ratio of outcome in intervention group relative to control group

**Table 4:** Summary of rurality adjusted results from logistic models for admissions to hospital and emergency department (ED) visits

**Outcome measure**	**Adjusted OR**^a^	**CI for OR**	***P*-value**
Admitted to hospital for any cause	0.70	0.54, 0.90	0.01
Admitted to hospital for Mental Health condition	0.43	0.19, 0.99	0.05
Admitted to hospital for COPD condition	0.57	0.30, 1.08	0.08
Admitted to hospital for diabetes condition	0.52	0.21, 1.30	0.16
Visited ED	0.80	0.62, 1.03	0.09

^a^*OR* Odds ratio of outcome in intervention group relative to control group

No statistically significant difference for the primary outcome, total length of stay, was found. Table 4 shows that the odds of being admitted to hospital (for any cause, and for each of the three specific causes), and the odds of visiting ED were lower for the intervention group. Only admission to hospital for any cause, and admission for mental health conditions were statistically significant. People in the intervention group had a 30% reduction in the odds of being admitted to hospital during the study year compared to those in the control group. They had 57% reduction in the odds of a mental health admission.

After adjusting for rurality, all the outcomes were more favourable for the intervention group than the control group, except for diabetes length of stay (Table 3). There were statistically significant differences in the number of admissions for mental health problems, admissions for COPD, and length of stay for COPD admissions. In the intervention group, the adjusted incidence rate ratio for mental health and COPD were 0.39 (CI: 0.169 to 0.915) and 0.37 (CI: 0.162 to 0.853) respectively, i.e. less than 40% of the control group. After adjusting for rurality, people in the intervention group only spent about 20% as many days in hospital for COPD as people in the control group.

## Discussion

The study shows that removing a small co-payment for medicines had a substantial and statistically significant effect on the odds of being hospitalised during the study year, reduced the number of admissions for mental health problems, the number of admissions for COPD, and the length of stay for COPD admissions. Although other outcomes (including the primary outcome) were not statistically significant, the rates of all negative outcomes were lower in the intervention group (except all-cause mortality and diabetes length of stay).

The key strength of the study is the comparison of a single simple intervention to usual care, mimicking a potential policy change and its incremental benefits. Additionally, although we did not recruit the sample size we aimed for, we were very successful in recruiting people facing socio-economic disadvantage and a significant burden of ill health [28]. The inclusion criteria were designed to identify the people most likely to be hospitalised because of cost-related nonadherence. We think they are representative of the group of people who face significant social disadvantage and have poor health, but they were not intended to be representative of the New Zealand population as a whole.

The key weakness was the smaller than anticipated sample size that reduced the power of the study to obtain statistically significant results. The much larger number of people in the intervention group than in the control group raises concerns about the randomisation, particularly if the control group had a higher burden of illness before the study. We have found no plausible explanation for the discrepancy in group size. One possibility might be that people withdrew their consent after being allocated to the control group, and we cannot rule out the possibility of this having happened on a very small number of occasions, not reported to us. However, this never happened in the case of either the call centre or the PI, who between them recruited more than half of the participants, and also obtained a greater number of people in the intervention group (see supplementary data). Although people may have been frustrated when they were allocated to the control group, they had nothing to lose by continuing in the study, and a $100 grocery voucher to gain in approximately 12 months time, so it seems unlikely that many would choose to drop out. A small number (less than 20) of enrolment forms were destroyed by one investigator before sending to the study team, because signed consent had not been obtained. It is likely (though not certain) that more of these were control group participants. We have been unable to find reports of problems with the technology we used for randomisation, which was set so that the memory was cleared after each use. The broad equivalency of the two arms across demographic characteristics and their very similar levels of medicines use in the previous year suggests that whatever led to the larger numbers in the intervention group was not systematic and did not affect randomisation.

The methods of recruiting may have limited the participation of people who were housebound and who had limited access to social media (although some were referred to us by family members). The presence of pharmacies waiving prescription charges in main urban centres meant the study was carried out in smaller cities and rural areas. The lack of centralised dataset of GP visits prevented any examination of the impact of the intervention on primary care utilisation. In line with the Ministry of Health’s definition, we identified hospitalisations from their administrative dataset of NMDS. ED visits of more than three hours duration are included in the hospitalisation data (i.e. they count as a hospitalisation, but, as with other hospitalisations, the length of stay is the number of midnights in hospital). The inclusion of these events in the primary outcome analyses might have been impacted by how busy the ED was, for example. However, we were able to examine ED visits specifically in a secondary analysis using the NNPAC dataset, which includes ED visits of any length.

The lack of a centralized system for ensuring people got the intervention (requiring us to rely on the cooperation of busy community pharmacy staff) is likely to have led to underestimation of the impact of the intervention because a small number of people were not exempted from co-payments if they forgot to take their study ID card to the pharmacy. The impact of the COVID-19 pandemic on the study is unclear. New Zealand had few cases during the study period and there was little impact on economic growth [41]. Some people experienced financial difficulties but at the same time government assistance was also increased. This could mean that more people in the control group received assistance with prescription co-payments than in a normal year, also leading to an underestimation of impact. Participants in the control group may have changed their behaviour in other ways, although low income is likely to have constrained their abilities to do this.

This study contributes to a body of literature showing that prescription co-payments are damaging to the health of vulnerable groups [19] and that they can increase use of other services especially when these services are provided without charge [21, 42]. To our knowledge, this is the first to provide evidence from an experimental study design, where the only change was to the price of prescriptions. In our study, participants continued to visit any pharmacy they chose, there was no change to the medicines they were prescribed, and prescribers were not made aware of their participation (unless participants chose to tell them).

The most likely explanation for our findings, that exemption from the $5 co-payment leads to improved outcomes, is that the $5 charge leads to people deferring or avoiding collecting medicines from the pharmacy which would have improved or maintained their health. Other possibilities include people in the control group avoiding primary care when they knew that they could not afford any medicines they might be prescribed, decreasing their consumption of other health-promoting goods (like food) in order to afford medicines, or reducing their dose of medicines so that they lasted longer [43, 44]. The mechanisms underpinning the differences in outcomes we observed between the intervention and control groups are likely to be complex. We will be exploring these potential mechanisms in a future paper.

The effect size appears to be very large compared with other studies. For example, the MI FREEE study reported a modest increase in adherence to cardiovascular medications for which copayments were waived and an 11% reduction in major vascular events or revascularization [27]. Tamblyn et al. [21] reported an increase of 6.8 serious adverse events (hospitalisation, nursing home admission, or mortality), per 10,000 person-months after the introduction of prescription cost-sharing in Quebec, Canada. However, both of these studies included large populations in which people presumably varied in income and health status. MI FREEE’s population were those insured with a private insurer in the US. In contrast, we deliberately selected participants who were at high risk of not being able to afford their medicines (because of high levels of socio-economic deprivation and health problems that frequently necessitate large numbers of medicines) and at risk of hospitalization if they went without their medicines [31–33]. The incomes of many of our participants are well below those needed for an adequate standard of living [45] so even a small amount of financial relief is likely to make a difference to their lives. In addition, in our study charges were waived for all medicines, not just those for one condition (as in MI FREEE).

Our study suggests that prescription co-payments are likely to increase overall healthcare costs. The cost of one day of hospital care in New Zealand has been estimated at $1000 [46] to over $1500 [47]. Increased ill-health caused by lack of access to medicines likely leads to other costs to government, other health and social care providers, and particularly to individuals and their families. In New Zealand it contributes to and exacerbates significant ethnic inequalities, because inability to afford medicines is much more common amongst Māori and Pacific people [13]. In addition, being unable to afford medicines is stressful [48] and erodes people’s mana (dignity) and social inclusion [43]. On the other hand, revenue generated by government from prescription co-payments is low. New Zealanders are exempted from the $5 charge after 20 items in a year, so revenue is, at most, $100 per person. Because the government indirectly pays some of the co-payments through the Disability Allowance and other schemes and bears much of the administrative costs of these and the 20 item exemption scheme, the net revenue generated must be significantly lower than $100 per person.

## Conclusion

The study strongly suggests that for people on low incomes, even small co-payments with a low ceiling can result in use of more expensive healthcare. In New Zealand, we strongly recommend that the $5 prescription co-payments be removed for those with high health needs and low incomes, or be scrapped entirely. The latter solution would be administratively simpler and avoid the risk that those with very high needs miss out because they do not successfully navigate bureaucratic processes [49]. It is likely that in other countries similar small charges have unanticipated negative consequences for health, for equity, and for health expenditure.

Further research should examine other small charges for healthcare that may have perverse consequences for healthcare funders and increase inequities. RCTs are expensive but not prohibitively so when the charges themselves are low.

## Supplementary Information

**Additional file 1:Supplementary Material.** ICD codes used for outcomes.

**Additional file 2.** Supplementary data on randomisation.

## Data Availability

The datasets generated and/or analysed during the current study are not publicly available because when we consulted with people who are similar to those in the trial, they were particularly sensitive about privacy and the confidentiality of their data. De-identified data however are available from the corresponding author on reasonable request.
