# Correction: Impact of removing prescription co-payments on the use of costly health services: a pragmatic randomised controlled trial

**DOI:** 10.1186/s12913-023-09251-8

**Published:** 2023-03-13

**Authors:** Pauline Norris, Kim Cousins, Simon Horsburgh, Shirley Keown, Marianna Churchward, Ariyapala Samaranayaka, Alesha Smith, Carlo Marra

**Affiliations:** 1 Va’a o Tautai- Centre for Pacific Health, University of Otago, PO Box 56, Dunedin, 9011 New Zealand; 2 Department of Preventive and Social Medicine, University of Otago, PO Box 56, Dunedin, New Zealand; 3 Turanga Health, 145 Derby St, Gisborne, 4010 New Zealand; 4 Health Services Research Centre, Victoria University of Wellington, PO Box 600, Wellington, New Zealand; 5 School of Pharmacy, University of Otago, PO Box 56, Dunedin, New Zealand

**Correction to**: *BMC Health Services Research* (2023) 23:31. 10.1186/s12913-022-09011-0.

Following publication of the original article [1], the authors identified some errors in the reported study years:

Corrections (marked in **bold**):

1. Participants were individually randomized (1–1 ratio) to either be exempted from the standard $5 charge per prescription item for one year (**2020–2021**) (n = 591) or usual care (n = 469). (Abstract under “Interventions”)
2. The primary outcome was length of stay (measured by hospital bed-days) within the year of the study **(1 Feb 2020 to 31 Jan 2021)** (Methods).

The original article [1] has been corrected.
