# Supplementary material for: Impact of removing prescription co-payments on the use of costly health services: a pragmatic randomised controlled trial
Source: BMC Health Serv Res. 2023 Jan 14;23:31. doi: 10.1186/s12913-022-09011-0 (PMC9839957; doi:10.1186/s12913-022-09011-0)
Supplement: Supplementary file 1 — Additional file 1:Supplementary Material. ICD codes used for outcomes. [file 12913_2022_9011_MOESM1_ESM.docx]

# Supplementary Material: ICD codes used for outcomes.

## Mental Health Admissions

Mental Health admissions were identified through a mixture of principal diagnosis codes for hospital admissions and ‘E’-coded admissions to identify self-harm events. The process was as follows:

1. For each admission, restrict to A- (principal) or E-type (external cause of injury) diagnoses.
2. Identify any diagnoses matching the ICD-10 codes referring to a target mental health event (see list).
3. Flag admissions with a mental health diagnosis identified in 2.

The table below lists the ICD-10 codes considered to refer to a mental health event:

| **ICD-10 Code** | **Description** |
| --- | --- |
| F* | Mental, Behavioural and Neurodevelopmental disorders (very broad!) |
| X6* | Intentional self-poisoning group |
| X7* | Intentional self-harm by object group |
| X80* | Intentional self-harm by jumping from a high place |
| X81* | Intentional self-harm by jumping or lying before a moving object group |
| X82* | Intentional self-harm by crashing of motor vehicle group |
| X83* | Intentional self-harm by other specified means |
| X84* | Intentional self-harm by unspecified means |
| Y870 | Sequelae of intentional self-harm |

## COPD Admissions

COPD admissions were restricted to hospitalisations where COPD was the principal diagnosis (and so excluded admissions for conditions such as pneumonia where COPD was listed in the secondary diagnoses). Only COPD was included; emphysema and chronic bronchitis were excluded.

Only one group of ICD-10 codes was used to identify COPD admissions:

| **ICD-10 Code** | **Description** |
| --- | --- |
| J44.0 | Chronic obstructive pulmonary disease with acute lower respiratory infection |
| J44.1 | Chronic obstructive pulmonary disease with acute exacerbation, unspecified |
| J44.8 | Other specified chronic obstructive pulmonary disease |
| J44.9 | Chronic obstructive pulmonary disease, unspecified |

## Diabetes Admissions

Diabetes mellitus (DM) admissions were restricted to hospitalisations where DM (type 1 or type 2) was the principal diagnosis (and excluded admissions for conditions where DM was listed in the secondary diagnoses). Only type 1 and type 2 DM were included; gestational diabetes was excluded.

Only two groups of ICD-10 codes were used to identify DM admissions:

| **ICD-10 Code** | **Description** |
| --- | --- |
| E10.1 | Type 1 diabetes mellitus with ketoacidosis |
| E10.2 | Type 1 diabetes mellitus with kidney complications |
| E10.3 | Type 1 diabetes mellitus with ophthalmic complications |
| E10.4 | Type 1 diabetes mellitus with neurological complications |
| E10.5 | Type 1 diabetes mellitus with circulatory complications |
| E10.6 | Type 1 diabetes mellitus with other specified complications |
| E10.7 | Type 1 diabetes mellitus, with unspecified complications |
| E10.8 | Type 1 diabetes mellitus, without complications |
| E10.9 | Type 2 diabetes mellitus with hyperosmolarity |
| E11.0 | Type 2 diabetes mellitus with ketoacidosis |
| E11.1 | Type 2 diabetes mellitus with kidney complications |
| E11.2 | Type 2 diabetes mellitus with ophthalmic complications |
| E11.3 | Type 2 diabetes mellitus with neurological complications |
| E11.4 | Type 2 diabetes mellitus with circulatory complications |
| E11.5 | Type 2 diabetes mellitus with other specified complications |
| E11.6 | Type 2 diabetes mellitus, with multiple complications |
| E11.8 | Type 2 diabetes mellitus, with unspecified complications |
| E11.9 | Type 2 diabetes mellitus, without complications |
