# Supplementary material for: Impact of removing prescription co-payments on the use of costly health services: a pragmatic randomised controlled trial
Source: BMC Health Serv Res. 2023 Jan 14;23:31. doi: 10.1186/s12913-022-09011-0 (PMC9839957; doi:10.1186/s12913-022-09011-0)
Supplement: Supplementary file 2 — Additional file 2. Supplementary data on randomisation. [file 12913_2022_9011_MOESM2_ESM.docx]

**Supplementary data on randomisation**

In these tables, recruiters who are study authors are identified by initials, while employees and students are anonymised.

Table 1: Percentage of participants in the intervention group by recruiter (in order of number of participants recruited)

|  | Intervention | Control | Grand Total | % Intervention | Excess intervention* |
| --- | --- | --- | --- | --- | --- |
| Call Centre | 189 | 160 | 349 | 54 | 14.5 |
| PN | 104 | 87 | 191 | 54 | 8.5 |
| Research Fellow# | 117 | 71 | 188 | 62 | 23 |
| KC | 89 | 68 | 157 | 57 | 10.5 |
| Student 4 | 34 | 21 | 55 | 62 | 6.5 |
| MC | 21 | 29 | 50 | 42 | -4 |
| SK | 22 | 22 | 44 | 50 | 0 |
| Student 1 | 7 | 5 | 12 | 58 | 1 |
| Student 3 | 1 | 4 | 5 | 20 | -1.5 |
| Student 2 | 3 | 1 | 4 | 75 | 1 |
| Grand Total | 587 | 468 | 1055 | 56 | 59.5 |

*Excess intervention = number in intervention group minus 50% of the total (i.e. the difference between the actual size of the intervention group and the expected size of the intervention group).

#This is the person who destroyed up to 20 enrolment forms, because participants did not come in to sign them. It seems likely that more of these were control group participants.

Table 2: Percentage of participants in the intervention group by those recruiting over 50 participants (in order of percentage of participants in the intervention group)

|  | Intervention | Control | Grand Total | % intervention | Excess intervention* |
| --- | --- | --- | --- | --- | --- |
| Research Fellow | 117 | 71 | 188 | 62 | 23 |
| Student 4 | 34 | 21 | 55 | 62 | 6.5 |
| KC | 89 | 68 | 157 | 57 | 10.5 |
| Call Centre | 189 | 160 | 349 | 54 | 14.5 |
| PN | 104 | 87 | 191 | 54 | 8.5 |
| SK | 22 | 22 | 44 | 50 | 0 |
| MC | 21 | 29 | 50 | 42 | -4 |

*Excess intervention = number in intervention group minus 50% of the total (i.e. the difference between the actual size of the intervention group and the expected size of the intervention group).
